# Is it just a cefazolin issue?

**DOI:** 10.1186/s40981-019-0281-7

**Published:** 2019-09-10

**Authors:** Tomonori Takazawa, Yukie Murooka, Shigeru Saito

**Affiliations:** Intensive Care Unit, Gunma University, 3-39-15 Showa-machi, Maebashi, Gunma 371-8511 Japan

## To the Editor,

The Ministry of Health, Labour and Welfare (MHLW) of Japan has recommended the use of generic medicines to reduce treatment costs [1]. In line with this recommendation, the switch from brand-name to generic medicines is progressing in Japan, with the latter accounting for 72.6% of all drugs sold in September 2018 on a volume basis [2]. Since the use of generic medicines is effective in controlling the rising medical costs, this is a favorable trend.

Recently, however, disruptions in medical practice have occurred as some of the prophylactic antibiotics that are often administered during surgery have been discontinued. For example, the supply of generic cefazolin (CEZ) has been suspended by a major generic drug company since February 2019. To cope with this, the MHLW announced a recommended list of CEZ alternatives at the end of March 2019 [3]. An online survey conducted by the MHLW in June 2019 showed restriction of CEZ use in almost half of the facilities that responded (474 out of 1071) due to the CEZ supply issue [4]. Although the drug company recently announced measures to secure multiple suppliers of the components of generic drugs, CEZ is only expected to be available after the fall of 2019 [5]. The sudden shift from CEZ to alternatives by many hospitals might lead to instability of the drug supply chain. Moreover, unnecessary use of broad-spectrum antibiotics might cause problems such as an increase in resistant bacteria. Apart from CEZ, some generics of ampicillin-sulbactam have also become unavailable since June 2018, and full recovery of the supply has not yet been achieved.

Given that companies are obliged to pursue profits in order to maintain investor satisfaction, expenditure for maintaining a stable drug supply is expected to be minimal. Ensuring patient safety during the perioperative period, including prevention of postoperative infection, is an extremely important goal of treatment. Hence, all individual medical staff members, including anesthesiologists, should consider a solution to this critical situation. Furthermore, representatives of each related academic society, pharmaceutical company, and the MHLW need to conduct negotiations to seek a stable supply of generic medicines. The prevalence of authorized generics produced by brand-name pharmaceutical manufacturers might be an option to solve this problem.

## Data Availability

Not applicable.

## Abbreviations

MHLW: The Ministry of Health, Labour and Welfare
CEZ: Cefazolin
